# One-year prevalence and the impact of migraine and tension-type headache in Turkey: a nationwide home-based study in adults

**DOI:** 10.1007/s10194-011-0414-5

**Published:** 2012-01-14

**Authors:** Mustafa Ertas, Betul Baykan, Elif Kocasoy Orhan, Mehmet Zarifoglu, Necdet Karli, Sabahattin Saip, Ayse Emel Onal, Aksel Siva

**Affiliations:** 1 Department of Neurology, Anadolu Health Center Hospital, Kocaeli, Turkey; 2 Department of Neurology, Istanbul Faculty of Medicine, Istanbul University, Istanbul, Turkey; 3 Department of Neurology, Faculty of Medicine, Uludag University, Bursa, Turkey; 4 Department of Neurology, Cerrahpasa Faculty of Medicine, Istanbul University, Istanbul, Turkey; 5 Department of Public Health, Istanbul Faculty of Medicine, Istanbul University, Istanbul, Turkey; 6 Anadolu Saglik Merkezi Hastanesi, Noroloji Klinigi, Cayirova, Kocaeli, Turkey

**Keywords:** Prevalence of migraine, Prevalence of tension-type headache, Migraine, Tension-type headache, Headache

## Abstract

Several studies have shown that the prevalence of migraine and tension-type headache (TTH) varied between different geographical regions. Therefore, there is a need of a nationwide prevalence study for headache in our country, located between Asia and Europe. This nationwide study was designed to estimate the 1-year prevalence of migraine and TTH and analyse the clinical features, the impact as well as the demographic and socio-economic characteristics of the participant households in Turkey. We planned to investigate 6,000 representative households in 21 cities of Turkey; and a total of 5,323 households (response rate of 89%) aged between 18 and 65 years were examined for headache by 33 trained physicians at home on the basis of the diagnostic criteria of the second edition of the International Classification of Headache Disorders (ICHD-II). The electronically registered questionnaire was based on the headache features, the associated symptoms, demographic and socio-economic situation and history. Of 5,323 participants (48.8% women; mean age 35.9 ± 12 years) 44.6% reported recurrent headaches during the last 1 year and 871 were diagnosed with migraine at a prevalence rate of 16.4% (8.5% in men and 24.6% in women), whereas only 270 were diagnosed with TTH at a prevalence rate of 5.1% (5.7% in men and 4.5% in women). The 1-year prevalence of probable migraine was 12.4% and probable TTH was 9.5% additionally. The rate of migraine with aura among migraineurs was 21.5%. The prevalence of migraine was highest among 35–40-year-old women while there were no differences in age groups among men and in TTH overall. More than 2/3 of migraineurs had ever consulted a physician whereas only 1/3 of patients with TTH had ever consulted a physician. For women, the migraine prevalence was higher among the ones with a lower income, while among men, it did not show any change by income. Migraine prevalence was lower in those with a lower educational status compared to those with a high educational status. Chronic daily headache was present in 3.3% and the prevalence of medication overuse headache was 2.1% in our population. There was an important impact of migraine with a monthly frequency of 5.9 ± 6, and an attack duration of 35.1 ± 72 h, but only 4.9% were on prophylactic treatment. The one-year prevalence of migraine estimated as 16.4% was similar or even higher than world-wide reported migraine prevalence figures and identical to a previous nation-wide study conducted in 1998, whereas the TTH prevalence was much lower using the same methodology with the ICHD-II criteria.

**Electronic supplementary material:**

The online version of this article (doi:10.1007/s10194-011-0414-5) contains supplementary material, which is available to authorized users.

## Introduction

Prevalence estimates of migraine as well as tension-type headache (TTH) show worldwide variations mainly due to the differences in the definitions and methodologies of the studies**.** It is remarkable, however, that the recent population-based studies in adults, all using the diagnostic criteria of the International Headache Society (IHS), have achieved similar prevalence rates of migraine. Several European [1–6] and American studies [7–9] have reported somewhat congruent prevalence figures about 10–12% for migraine in adults, 6% among men and 15–18% among women. A meta-analysis indicated that the prevalence of headache and migraine varied between different geographical regions, being somewhat lower in Europe than in North America but higher than in Asia and Africa [10]. So, there is a need for independent prevalence studies of migraine in different regions of the world using the IHS criteria.

The prevalence of TTH varied much more widely among studies, and more attention has been drawn to its importance during the last years. Even though TTH was known to be the most prevalent type of headache across all age groups worldwide [11, 12], there were still relatively few epidemiological studies on TTH. Authors reported that it was a paradox that the prevalence of TTH seemed higher than that of headache in general in the European studies [6]. Therefore, there is a second need for epidemiological studies investigating the TTH prevalence by strictly using the IHS criteria.

Although there were a few local epidemiological studies on headache [13–16] in our country located between Asia and Europe, published nationwide studies assessing the headache in adults are lacking. In a preliminary nationwide population-based headache survey in Turkey conducted in 1998 using the criteria of first edition of the International Classification of Headache Disorders (ICHD-I, 1988) [17], migraine prevalence was estimated to be 16.4% and TTH 31.7% among 2,007 households aged between 15 and 55 years [18].

We aimed to investigate the nationwide migraine and TTH prevalence and analyse the clinical features as well as the demographic and socio-economic characteristics using the second edition of the ICHD (ICHD-II, 2004) criteria [19] for the first time in a large sample using a population-based design in Turkey.

## Methods

We designed a nationwide, community- based prevalence study in adults aged between 18 and 65 years, with face-to-face interviews by 33 specially trained general practitioner physicians using a structured electronic questionnaire. The comprehensive interview form included diagnostic questions based on the ICHD-II criteria [19] and revised criteria for chronic migraine and medication overuse headache (MOH) [20] for diagnoses of migraine, TTH and MOH within the last 1 year, questions about features of headache and associated symptoms, demographic and socio-economic conditions of the participants, information about the previous physician visits, previous diagnoses, disability assessment by Turkish version of MIDAS questionnaire [21], acute and prophylactic medication in migraineurs. TTH was diagnosed if the participants were not diagnosed with “definite” or “probable” migraine and fulfilled all TTH criteria.

We used a multi-stage sampling strategy, which involved as the initial stage, the selection of 21 cities representative of the characteristics of households in all 7 geographical regions of Turkey based on the ratio of their population to the total population of Turkey as reported in the year of 2008 by the Turkish Statistical Institute (http://rapor.tuik.gov.tr/reports/rwservlet?adnksdb2=&ENVID=adnksdb2Env&report=turkiye_yasgr.RDF&p_yil=2008&p_dil=1&desformat=html). Six of the seven geographical regions of Turkey were each represented by three different cities and only one last smallest region was investigated by two representative cities. As the largest city of Turkey with a high internal migration rate, Istanbul was considered as a different region and represented by 1,400 households. In the second part of selection, the distribution of urban and rural populations, gender and age groups were all taken into account to choose the target population in these cities, to ensure that there will be no selection bias. The total population of Turkey with an age range of 18–65 years which was around 40 million was represented by 6,000 households with an acceptable error rate of ±1.3%. After establishing the total number of households to be interviewed (for example *n* = 240 households for the city of A), this number was further divided by the urban and rural populations of this specific city. By the guidance of the quotas for each city, the houses to be visited were determined using a simple random-sampling method in districts, streets and rural areas. Only one person was interviewed in each household to avoid any bias. A Kish sampling grid was used to select one person per household to be interviewed. A total of 6,000 households were visited. After excluding the households visited but not interviewed because of several reasons such as (“rejecting to be interviewed”, “having no time”, “non-presence at home” etc.), 89% of the households had valid interviews. At the end, the statistical standard error was ±1.3% within 95% confidence interval for 5,323 interviews, as planned.

The study was completed within 3 months in the year of 2008. Each of the 33 physicians visited 1–3 cities and each city was visited by 1–10 physicians. In every visited home, a physician accompanied by an interviewer administered the questionnaire using an electronic palm device connected to study-headquarters by mobile phone card to transfer the data online.

The role of the physician was to exclude secondary headaches and medical conditions interfering with the primary headache disorders, by examining the patients and reviewing their related investigations such as MRIs, LP records, sinus radiographs, etc. were available. They interviewed the participants about the previous physician visits related to headache in detail, discussed about the diagnoses established and reviewed all the available medical reports. For example if a participant reported that he had the diagnosis of sinusitis, the history about acute and chronic presentations and temporal relationship with headache attacks were ascertained after viewing the radiographs.

The headache diagnosis was based on the answers of the questionnaire, according to the ICHD-II criteria. We used the 1-year prevalence figures, indicating the proportion of the population that had an active disease, which was more relevant than the lifetime prevalence, which was considered less reliable due to recall problems. The age groups below 18 years (children) and over 65 years (elderly) were not included in investigation of the prevalence rate in adults.

The questionnaire assessed the headache features, diagnosis, headache related impact, demographics, and disability assessed by the Turkish version of MIDAS questionnaire. The participants were asked to provide the mean number of attacks and mean number of days with headache per month during the last year and the untreated duration of attack in hours. Aura was described as the recurrent symptoms starting before or just with the start of the headache lasting 5 min to 1 h. Five types of aura namely: visual (hemianopia and flashing lights) somatosensory, speech disturbances, vertigo/dizziness and motor dysfunction were questioned separately.

Descriptive statistics were applied and Chi-square test, *t* test and logistic regression test were used for the group comparisons, where appropriate. We used the SPSS 15 software.

## Results

A total of 5,323 participants (2,600 women and 2,723 men) were reviewed. Of the study population, 82.8% are city dwellers, 16.4% are borough dwellers and 0.8% are village dwellers. The ages of participants ranged between 18 and 65 years with a mean of 36.2 ± 12 years for women and 35.7 ± 12 years for men. These distributions of participants are comparable to the demographics of Turkey as reported in the year of 2008 by the Turkish Statistical Institute.

### Migraine prevalence

**Fig. 1:**
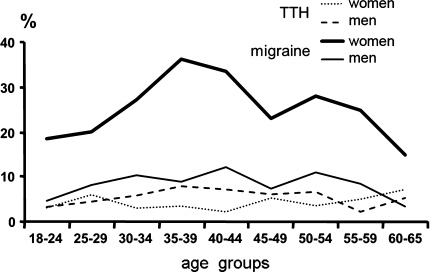
Migraine and tension-type headache prevalence in age groups in relation to gender

2,376 (44.6%) participants reported recurrent headaches within the last 1 year, whereas 2,947 were free of recurrent headaches. Of these 2,376 participants with headache, 1,373 (57.8%) were women and 1,003 (42.2%) were men. Of the total study population, 871 were diagnosed with “definite” migraine and the 1-year prevalence of migraine was estimated to be 16.4%. The details of prevalence of definite and probable migraine diagnosed based on the ICHD-II criteria by gender are shown in Table 1. The rate for migraine with aura among migraineurs is 21.5%. The prevalence of migraine is highest among 35–40-year-old women while there is seemingly no such great difference in age groups among men (Fig. 1).

**Table 1:** The prevalence of migraine types according to gender in Turkey

	Women, *n* = 2,600 (%)	Men, *n* = 2,723 (%)	Total, *n* = 5,323 (%)
Definite migraine	640 (24.6)	231 (8.5)	871 (16.4)
Probable migraine	349 (13.4)	313 (11.5)	662 (12.4)
Migraine with aura	135 (5.2)	52 (1.9)	187 (3.5)
Probable migraine with aura	42 (1.6)	45 (1.7)	87 (1.6)
Chronic migraine	17 (0.7)	6 (0.2)	23 (0.4)
Probable chronic migraine (with medication overuse)	56 (2.2)	15 (0.6)	71 (1.3)
Total migraine (definite + probable)	989 (38.0)	544 (20.0)	1,533 (28.8)

### TTH prevalence

After excluding the participants who were diagnosed with “definite” or “probable” migraine, 270 of the remaining were diagnosed with “definite” TTH according to the ICHD-II criteria and the 1-year prevalence of “definite” TTH was estimated to be 5.1%. All details of the TTH prevalence regarding rare episodic, frequent episodic and chronic TTH by gender are presented in Table 2. Figure 1 also shows the percentage of the patients with TTH within the age groups of the study population, which did not show any significant difference by gender.

**Table 2:** The prevalence of tension-type headache (TTH) types according to gender in Turkey

	Women, *n* = 2,600 (%)	Men, *n* = 2,723 (%)	Total, *n* = 5,323 (%)
Definite TTH	116 (4.5)	154 (5.7)	270 (5.1)
Rare episodic	81 (3.1)	94 (3.4)	175 (3.3)
Frequent episodic	34 (1.3)	50 (1.8)	84 (1.6)
Chronic	1 (0.04)	10 (0.4)	11 (0.2)
Probable TTH	228 (8.8)	276 (10.1)	504 (9.5)
Rare episodic	157 (6.0)	213 (7.8)	370 (6.9)
Frequent episodic	66 (2.5)	52 (1.9)	118 (2.2)
Chronic	5 (0.2)	11 (0.4)	16 (0.3)
Total TTH (definite + probable)	344 (13.2)	430 (15.8)	774 (14.5)

### Unclassified headache

Total 69 patients (1.3% of study population) had reported other types of recurrent headaches not diagnosed as definite or probable migraine or TTH. Of these 69 patients with unclassified headache, 64 (1.2%) had episodic headache and 5 (0.09) had chronic headache.

### Physician consults and headache diagnoses

**Fig. 2:**
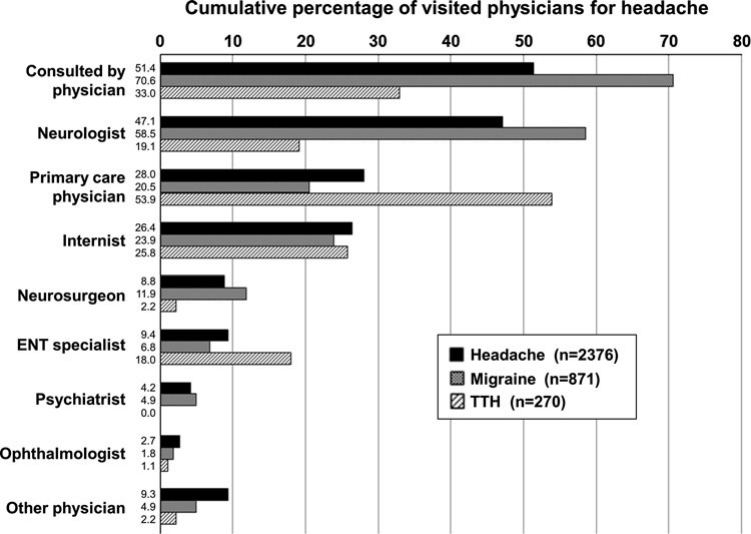
Cumulative percentage of visited physicians for headache. (For example, of 2,376 headache sufferers, 47.1% consulted neurologist)

The analysis of physician consults for headaches revealed that more than two-thirds (70.6%) of migraineurs had consulted a physician, whereas only one-third of the TTH patients had a physician visit, with a significant difference between the headache groups. Mostly consulted physicians were neurologists as seen in Fig. 2. Previous headache diagnoses of patients with migraine and TTH are outlined in Table 3. In the analysis of migraineurs for previous diagnosis of their migraine headaches, less than half had diagnosis of migraine (42.0%) at the first physician visit and only half of migraineurs (51.2%) had diagnosis of migraine at the first or the following visits. Misdiagnoses included TTH (or psychogenic headache), sinusitis, hypertension, cervicogenic headache, and headache due to vision problem, in order of decreasing frequency. One-third of TTH patients were misdiagnosed with sinusitis, followed by other misdiagnoses such as hypertensive or cervicogenic headache, but definite pure TTH patients diagnosed with migraine were really rare (1%).

**Table 3:** Previous headache diagnoses of migraineurs and patients with TTH

	Patients with definite migraine		Patients with definite TTH	
	First Dx of migraine, *n* = 615^a^ (%)	Cumulative Dx of migraine, *n* = 615^b^ (%)	First Dx of TTHs, *n* = 89^a^ (%)	Cumulative Dx of TTHs, *n* = 89^b^ (%)
Migraine	42.0	51.2	1.1	1.1
Tension/psychogenic	22.8	30.1	33.7	43.8
Sinusitis	15.3	18.9	37.1	39.3
Hypertension	3.6	4.1	10.1	11.2
Cervicogenic	2.6	3.9	5.6	9.0
Vision problem	1.0	1.0	0.0	1.1
Other	12.8	14.1	12.4	12.4

*Dx* diagnosis, *TTHs* tension-type headache patients

^a^Diagnosis of first physician (one participant has one diagnosis)

^b^Cumulative percentage of diagnoses made by first and other physicians (one participant might have more than one diagnosis)

### Socio-economic characteristics

Tables 4 and 5 summarize certain socio-economic characteristics of the study population**.** Migraine prevalence is higher among unemployed for both genders and housewives. In women, migraine prevalence is higher (26.4%) among the ones with a lower income (less than 1,300 US$ monthly) than the ones with a higher income (20.3%), while in men, it is the same in the ones with lower income (8.5%) and the ones with higher income (8.5%). There is no change in prevalence by income in patients with TTH, in both genders (Table 5). Regarding the educational status of participants, migraine prevalence is highest among illiterates (31.6%) while it is 20.4% among participants who could read and write only without formal education, 19.1% among primary school graduates (5-year education), 14.2% among junior high school graduates (8-year education), 15.0% among high school graduates, and 14.9% among university graduates. Thus, migraine prevalence is lower in those with a lower educational status than those with a high educational status.

**Table 4:** Some socio-demographic characteristics of participants and comparison between headache types

	Study group (%)			Migraine group (%)			TTH group (%)		
	Women (*n* = 2,600)	Men(*n* = 2,723)	Total (*n* = 5,323)	Women (*n* = 640)	Men (*n* = 231)	Total (*n* = 871)	Women (*n* = 116)	Men (*n* = 154)	Total (*n* = 270)
University degree	22.8	23.9	23.4	19.5	26.8	21.5	20.7	25.3	23.3
Housewife/unemployed	48.9	7.6	27.9	58.1	13.4	46.3	53.4	5.8	26.3
City dweller	84.8	80.9	82.8	81.3	84.0	82.0	86.2	77.3	81.1
Monthly income <1,300 US$	70.5	75.0	72.8	75.6	74.9	75.4	71.6	76.6	74.4
House owner	66.2	638	65.0	61.9	64.9	62.7	64.7	59.7	61.9

**Table 5:** Economical profile of participants and migraine prevalence in relation to income groups

	Migraine prevalence (%)			TTH prevalence (%)		
	Women (*n* = 640 of 2,600)	Men (*n* = 231 of 2,723)	Total (*n* = 871 of 5,323)	Women (*n* = 116 of 2,600)	Men (*n* = 154 of 2,723)	Total (*n* = 270 of 5,323)
Living area						
Metropolitans	24.1	8.1	16.2	4.7	5.6	5.1
Smaller cities or areas	26.1	9.3	16.7	3.8	5.8	4.9
*P* value	NS	NS	NS	NS	NS	NS
Income (monthly)						
<1,300 US$	26.4	8.5	17.0	4.5	5.8	5.2
≥1,300 US$	20.3	8.5	14.7	4.3	5.3	4.8
*P* value	0.000	NS	0.028	NS	NS	NS

*P* value in Chi-square test

### Chronic daily headache and medication overuse headache

Medication overuse headache (MOH) was found in 114 (2.1%) of the total study population according to the revised criteria of MOH [20], being in 8.2% of patients with migraine, whereas this figure was 1.9% among patients with pure TTH. Chronic daily headache was diagnosed in 3.3% of the study population. Prevalence rate is 1.8% for chronic migraine (0.4% for those without medication overuse and 1.3% for those with medication overuse), and 0.2% for chronic TTH (0.1% for those without medication overuse and 0.09% for those with medication overuse). Chronic daily headache was present in 10.9% of definite migraineurs, 7.1% of probable migraineurs and 4.1% of definite TTH patients, and 3.2% of probable TTH patients.

### Attack characteristics

We further investigated the attack characteristics, disability, and the medication history in migraine sufferers by gender. Regarding the headache characteristics in migraineurs (Table 6), the average attack number was nearly 6 per month lasting nearly 1.5 days per attack. Attack durations tended to be shorter in men when compared to women and women experienced more nausea and allodynia compared to men (Table 6). More than half of the migraineurs (54.2%) reported that their headache attacks were usually severe. Of migraineurs who never sought medical advice, 40% had severe headache whereas 60% of those who ever consulted had severe headache. Of migraineurs, 54.5% reported headache limited to one side (persistently at one side or side-shift from attack to attack), 72.9% reported headache limited to or predominant on one side. In 27.1% of migraineurs, headache was always equal in both sides.

**Table 6:** Attack characteristics of migraineurs

	Women	Men	*P* value	Total
Number of attacks per month (mean ± SD)	6.0 ± 6^d^	5.7 ± 6	NS^a^	5.9 ± 6
Attack duration in hours	39.2 ± 78	24.0 ± 49	0.002^a^	35.1 ± 72
(mean ± SD)				
Headache days per month	6.3 ± 6	6.0 ± 6	NS^a^	6.2 ± 6
(mean ± SD)				
≥15 headache days per month (%)	11.4	9.5	NS^b^	10.9
Headache severity (%)				
Usually mild	5.8	9.5	NS^c^	6.8
Usually moderate	39.8	36.8		39.0
Usually severe	54.4	53.7		54.2
Limited to one side	52.2^d^	61.0	0.012^b^	54.5
Throbbing	83.4	76.6	0.015^b^	81.6
Increase with activity	93.8	92.6	NS^b^	93.5
With nausea or vomiting	84.4	71.0	0.000^b^	80.8
With photophobia	82.7	82.3	NS^b^	82.5
With phonophobia	85.8	83.5	NS^b^	85.2
With photo- and phonophobia	77.0	77.1	NS^b^	77.0
With allodynia	64.1	52.8	0.002^b^	61.1

^a^In unpaired *t* test

^b^In Chi-square test

^c^In logistic regression test

^d^Mean ± SD

In the disability assessment of the migraineurs, a MIDAS score of 1 (none or minor disability due to migraine) was reported in 54.9% of migraineurs while the score was 2 (mild disability) in 19.7%, the score was 3 (moderate disability) in 15.8% and finally, the score was 4 (severe disability) in 9.5%.

### Attack medication

As an attack medication, 19.3% of 871 migraineurs reported the use of simple analgesics, 15.8% combined analgesics, 41.4% nonsteroidal anti-inflammatory drugs (NSAIDs), 14.5% ergots, and only 2.9% triptans. MOH was found in 8.2% of migraineurs (8.8% in women and 6.5% in men). Overused medications were simple analgesics alone in 4.8% of migraineurs (5.3% in women and 3.5% in men) or combinations of ergots, triptans and analgesics in 3.3% of migraineurs (3.4% in women and 3.0% in men). Only in 43.1% of migraineurs, medication advice was given by physician. Chronic migraine without MOH was diagnosed in 2.6% of migraineurs (2.7% in women and 2.6% in men).

### Prophylactic medication

Although more than half of the migraineurs reported usually severe headache attacks and 4 or more attacks per month, only 4.9% were on prophylactic medication with mostly antidepressants (3.9% at the time of the questionnaire). Mostly used antidepressants were selective serotonin reuptake inhibitors (SSRIs) with a current rate of 2.8% and tricyclics with 1.0%. The current and past use of other prophylactic treatments such as beta blockers, flunarizin and antiepileptic drugs were less than 1% each.

## Discussion

Our nationwide population-based study estimated the 1-year prevalence of definite migraine as 16.4%, probable migraine as 12.4% and of pure TTH as 5.1%, probable TTH as 9.5% with ICHD-II criteria, constituting a total of 43.4% of the general population suffering from these two primary headache types. We had planned to reach 6,000 representative households and in the end, a total of 5,323 households were examined for headache. This excellent response rate of 89% probably reflects the conductance of the study directly by physicians face-to-face rather than sending a questionnaire. The prevalence of migraine was highest among 35–40-year-old women while there were no big differences in age groups among men and in TTH overall, as shown in Fig. 1.

The striking well-known female preponderance in patients with migraine which is also evident in our study is more consistent across studies than the overall prevalence figures of migraine [1, 5, 14]. All of the studies reveal that migraine is [6] two or three times more common in females than in males. Interestingly, the rates of the present study using ICHD-II criteria for migraine in adults aged 18–65 years (16.4%) as well as for migraine aura (21.5% in migraineurs) are identical with the previous largest Turkish nation-wide headache prevalence study with the participation of 2,007 households aged between 15 and 55 years [18] with the ICHD-I criteria. Some studies indicate that the prevalence of headache and especially of migraine has been increasing during the last decades in Europe [6, 22, 23]. Although our study showed no significant change in the migraine prevalence compared to the national study of 10 years ago from the present study, the male to female ratio was 1:3 in the present one while it was 1:2 in the previous one. Our study with more than the double sample size in comparison to the former one probably reflects the real gender difference. Although it is well-established that headache suffering, including migraine, was highly prevalent especially in younger women overall in the world, the differences of headache features between men and women were thoroughly investigated only in a few studies [24]. Our study showed that women had a significantly longer attack duration, more nausea and more allodynia in comparison to men among other differences as seen in Table 6. A population-based study in the UK reported the mean headache duration of 28.4 h in men versus 36.7 h in women along with non-significant changes of attack frequency and pain intensity, similar to our results [5]. Several hypotheses have been proposed to explain these differences, including fluctuations in sex hormones and receptor binding, genetic factors, differences in exposure to environmental stressors, as well as differences in response to stress and pain perception [24].

On the contrary of the small changes in the migraine prevalence around the world, the prevalence of TTH is a matter of debate and has varied widely among studies. TTH is known as the most prevalent type of headache across all age groups worldwide [11]. Nineteen studies have reported the TTH prevalence in Europe and the prevalence of current TTH among 66,000 adults was reported as 62.6%, and chronic TTH (i.e. on 15 days per month) occurred in 3.3%. Much lower figures (current TTH 15.9%, chronic TTH 0.9%) were found in the nine studies among almost 25,000 children and the youth showing the possible increase with age [6, 23]. The largest American study with telephone surveys reported a TTH prevalence of 38.3% [11, 25] and higher figures and lifetime prevalence around 80% were reported in Denmark [26]. In our study, TTH prevalence is much lower than most of the other studies, even after the inclusion of cases with probable TTH, interestingly. Rare episodic form is the most frequent form of TTH and followed by frequent episodic form and lastly chronic TTH is the most infrequent form in both definite and probable TTH categories, in our study.

The wide variations in the estimated prevalence of TTH can result from the methodology, case definitions, sampling procedures, possible influence of the physician/investigators and the inclusion or exclusion of cases of infrequent episodic TTH and overlap with probable migraine. We applied the ICHD-II 2004 criteria very strictly, without allowing any influence of the physician. It is also highly likely that some unknown genetic factors besides variables such as environmental risk factors or culturally determined differences in symptom reporting may further explain this discrepancy. It is important to note that in this study, TTH was a diagnosis of exclusion and it was only diagnosed in headache sufferers if definite or probable migraine were not diagnosed according to ICHD-II criteria. Hence, this could be one of the important reasons that the TTH rate in our study is not as high as the previous study in our country [18].

The difference of results between these two Turkish headache epidemiological studies can also be evaluated, considering the continuum hypothesis as a basis. The two ends of headache spectrum are TTH and migraine, both might evolve into other during time or from one attack to another. Mixed headache, so called TTH and migraine in the same individual, is accepted as the occurrence of spectrum of headache in the same individual [27]. Both adolescent and adult studies have shown that headache might evolve into both ends of spectrum [28, 29]. Thus, the low prevalence of TTH might be the evidence of evolving of TTH into probable migraine/migraine by some external or internal modifiers such as socio-economic difficulties or hormonal changes.

Another alternative conceptual approach, the “severity model” of headache, considers a continuum of headache ranging from mild to severe forms with specific headache subtypes distinguished by level of severity rather than unique constellations of symptoms [30].

Stovner and Colette [6] compared the results from the studies using different methods of data collection and reported that only for migraine and headache in general could meaningful comparisons be made; in relation to TTH, there were too few studies available. Most questionnaire studies use somewhat modified criteria, whereas studies based on personal interviews seem to give somewhat higher prevalence than those using questionnaires. The ways the ICHD criteria are applied and the diagnoses included are also of great importance. The problem of multiple headache types occurring in the same patient may represent problems in headache epidemiologic studies. One diagnostic dilemma is the overlap between TTH and probable migraine. It is well-known in clinical practice that many patients have comorbid TTH and migraine, or in other words many migraineurs may experience headaches very similar to TTH in some of their attacks. Thus, the trend and thoughts of the physician could affect the diagnosis. Being aware of this, our study was based on the strict computerized application of ICHD-II criteria aiming to exclude the subjectivity of the conducting physicians. Furthermore, some individuals suffer from infrequent, not disturbing headaches and could not remember the exact profile. It is also known that subjects’ headache symptoms might change during a given period or they might even forget that they had experienced headache [29]. All these factors pose difficulties in diagnosing headache in the population based epidemiological studies. This is particularly true for the probable headache diagnoses. Using ICHD-II criteria strictly, we showed that pure TTH is indeed rare. In ICHD-II, fulfilment of the diagnostic criteria for main groups of migraine and TTH or any of their subtypes, always trumps fulfilment of criteria for the probable diagnostic categories [19].

Although many studies investigated the prevalence of migraine and TTH in Western Europe and North America, there are only a few studies carried out in Eastern Europe. In the Republic of Georgia, an eastern neighbour of our country, one-year prevalence was estimated to be 6.5% for migraine, 9.2% for probable migraine (all migraine 15.6%), 10.0% for TTH, 27.3% for probable TTH (all TTH 37.3%) in a community-based door-to-door survey, conducted by four medical residents [31]. So they found a lower rate for migraine but a higher rate for TTH in comparison to our results. Another study from Croatia located also in the eastern bank of Europe reported a crude and lower prevalence of TTH as 21.2% [32]. It is interesting to note that both of these studies also showed relatively low prevalence rates of TTH, like in our study. Whether these regional differences are real or mainly a result of differences in the methodology and conduction of the studies is uncertain.

The prevalence of chronic daily headache (≥15 headache days per month) was 3.3% in our nationwide study, similar to many studies worldwide [12, 33–36]. Interestingly, an unusually high prevalence of chronic headache with a rate of 7.6% was reported from Georgia associated with a low socioeconomic status [31], showing variability of headache disorders, even in neighbours. Another population-based study from Far East of chronic daily headache in 3,377 participants reported a prevalence of 3.2% being higher in women (4.3%) than men (1.9%) similar to our results [35]. A 2.1% prevalence rate for MOH in our total study population seems to be some higher than reported rates before [31, 35, 37–39], however, recent studies reported higher rates of MOH in general population as in our study [34, 40, 41]. A reason for high rate of MOH in our study population might be related with low rate of prophylactic medication use which is 4.9% among migraineurs.

Although migraine is a remarkably common cause of temporary disability worldwide, many migraine sufferers have never consulted a physician. While 47.0% of migraineurs had physician consult for their headache in 1998 [18] in Turkey, this ratio has raised to 70.6% in 10 years. Though consultation rates have increased remarkably the underlying epidemiology of migraine remains stable over a decade in our country. Thus, our data support that there is no evidence of increasing prevalence of migraine with increased awareness. On the other hand, only one-third of the TTH patients had ever consulted a physician in 2008. Mostly consulted physicians were neurologists as seen in Fig. 2. Primary care physicians, who are supposed to be the first to consult for headache, were far less than neurologists in our country, reflecting the choice of the patients. A study from United States reported that 66.1% of migraineurs (68.1% in females and 57.3% in males) had ever consulted a physician [42]. While in this American study 61% of migraineurs who never consulted reported severe headache, in our study 40% of migraineurs who never consulted had usually severe headache. Of migraineurs who never consulted, 47.6% had 4 or more attack frequency per month, 14.1% had more than 1.5 days average attack duration and 21.5% had more than 6 headache days per month whereas of those who ever consulted, 57.4% had 4 or more attack frequency per month, 28.4% had more than 1.5 days average attack duration and 36.4% had more than 6 headache days per month. These facts reflected that there were still some patients with significant impact of migraine who did not consult for their headaches.

Prevalence studies exploring the relation between socio-economic status (SES) and headache have shown some conflicting results. The present study revealed a negative correlation of migraine prevalence with educational status unrelated to gender and with socioeconomic status only in women. Higher prevalence with lower educational status/lower income was reported in some other studies [1, 10, 42–44]. This contradicts the usual clinical perception that migraine is a disease of rich people. In previous studies done in Turkey, there was a positive correlation showing higher migraine prevalence with higher educational status [13, 45, 46]. These studies are possibly reflecting that people with higher income/education are far more likely to consult a physician or volunteer to participate in a study. In three very large population based studies in United States, the decline of migraine prevalence with increased income or education has been explained by “social causation hypothesis” such as “factors with low socioeconomic status increase migraine prevalence” and “social selection hypothesis” such as “migraine-related dysfunction interferes with educational and occupational functioning leading to low income and low education” [42–44]. A prospective study analysing the relation between SES and risk of headache in Norway showed that low SES was associated with increased risk of frequent and chronic headache at follow-up [47]. Interestingly, the risk of frequent and chronic headache decreased with increasing individual income, but only among men [47], showing again a gender difference of SES with migraine.

Every type of misdiagnosis is still very common both for migraine and TTH in our country as shown in Table 3. Furthermore, prophylactic medication usage was unexpectedly low (4.9%), even though neurologists were in charge for headache care for most of the patients. These points draw attention to the need of continuing education for headache management for physicians and for public to lift the real burden. The headache lectures and courses addressed mostly secondary headaches in the medical curriculum and seemed not be sufficient for appropriate management of primary headaches, taking the overall burden in daily life into account. Moreover, the optimal visit duration of headache patients should not be short. This is one of most limiting problems of Turkish neurologists who should examine huge numbers of patients every day.

There are some strong points of our study including face-to-face assessment of headaches by a specifically trained physician group with electronic database system, a large nation-wide sample size and a random population, strict application of the ICHD-II diagnostic criteria of the IHS excluding the subjectivity of the physician’s diagnosis. However, there is an unavoidable risk of the effect of the question style even with the same questions and with an electronic recording system. Due to the higher impact of migraine in clinical practice in our country [45] it is possible that the physicians are more prone to handling the migraine patients than the TTH sufferers.

In conclusion, our study showed a 16.4% prevalence rate of migraine in Turkey, and it is similar or even higher than the well-established prevalence figures of migraine worldwide. Although there are still misdiagnoses, the rate of physician consults for migraine has remarkably increased to 70.6%, whereas the rate of migraineurs on prophylactic treatment is still lower than expected. Finally, the prevalence of TTH with strict application of the 2004 ICHD-II diagnostic criteria is very low in our study (5.1% for definite TTH and 9.5% for probable TTH), a finding which could reflect some unknown genetic, cultural, environmental factors or methodological differences in the study designs.

## Electronic supplementary material

Below is the link to the electronic supplementary material.

Appendix 1 (PDF 6 kb)

Appendix 2 (PDF 24 kb)

## Abbreviations

TTH: Tension-type Headache
IHS: International Headache Society
ICHD-I: First edition of the International Classification of Headache Disorders
ICHD-II: Second edition of the International Classification of Headache Disorders
