# Supplementary material for: One-year prevalence and the impact of migraine and tension-type headache in Turkey: a nationwide home-based study in adults
Source: J Headache Pain. 2012 Jan 14;13(2):147–57. doi: 10.1007/s10194-011-0414-5 (PMC3274583; doi:10.1007/s10194-011-0414-5)
Supplement: Supplementary file 1 — Appendix 1 (PDF 6 kb) [file 10194_2011_414_MOESM1_ESM.pdf]

Appendix 1

- Study subjects questioned
- Age
- Gender
- Socioeconomic condition
- Allergy and asthma
- Vertigo/dizziness
- Depression
- Headache history of last 1 year
- Physician consultation for headache
- Diagnoses of headache
- Headache medication history
- Migraine aura
- Allodynia
- Lifetime history of headache
- Headache attack duration, frequency of monthly headache attacks and headache days
- Headache localization
- Features of headache and associated symptoms
- Headache severity
- Current headache diagnosis
- Acute medication history for headache, frequency of daily and monthly acute medication
- Response to acute medication
- Excessive use of acute medication
- Prophylactic headache medication history
- Disability due to headache
