# Supplementary material for: One-year prevalence and the impact of migraine and tension-type headache in Turkey: a nationwide home-based study in adults
Source: J Headache Pain. 2012 Jan 14;13(2):147–57. doi: 10.1007/s10194-011-0414-5 (PMC3274583; doi:10.1007/s10194-011-0414-5)
Supplement: Supplementary file 2 — Appendix 2 (PDF 24 kb) [file 10194_2011_414_MOESM2_ESM.pdf]

Appendix 2

- Questions
- Your Full name
- Your phone number
- Your sex
- Your date of birth (day) (real date of birth)
- Your date of birth (month)
- Your date of birth (year)
- Your age
- Your place of birth (city)
- Your height
- Your weight
- Your marital status
- Your educational status
- Your profession or job status
- Please state where you live

  1. Metropolis
  2. City
  3. Suburban district
  4. Town
  5. Village/settlement

- Please state the city you live
- Please state how long you have been living here
- Is there any city you lived before?
- Please state the city you lived before
- Please state how long did you live there
- Is there another city you lived before that?
- Please state that city
- Please state how long did you live there
- Please state your monthly income level (of your household)

  1. 0–499 TRY
  2. 500–999 TRY
  3. 1000–1999 TRY
  4. 2000–2999 TRY
  5. 3000–3999 TRY
  6. 4000–4999 TRY
  7. 5000–10.000 TRY
  8. Above 10.000 TRY

- Please indicate the applicable options for you

  1. Owns his house
  2. Has a car
  3. Has a computer
  4. Has a washing machine
  5. Has a dishwasher
  6. Has a refrigerator

- Your social security:

  1. Retirement fund
  2. Social security for artisans and self-employed
  3. Social security institution
  4. Private health insurance
  5. Green card
  6. Unsecured—paying externally

- Please state how many people live in your house
- “NOTE: Please indicate the relevant option based on your own judgment without asking to the respondent!”

  Socio-economic status:

  1. High income group
  2. Medium–high income group
  3. Middle class
  4. Low income group

- Your address
- Your city
- Region will be indicated.

  1. Istanbul
  2. Marmara Region (Edirne, Kırklareli, Bursa)
  3. Aegean Region (İzmir, Denizli, Kütahya)
  4. Mediterranean Region (Adana, Mersin, Antalya)
  5. Central Anatolian Region (Ankara, Konya, Kayseri)
  6. Black Sea Region (Zonguldak, Samsun, Trabzon)
  7. Eastern Anatolia Region (Erzurum, Malatya, Van)
  8. Southeastern Anatolia Region (Diyarbakır, Gaziantep)

- Do you smoke?
- If you quit smoking how long has it been?
- If you smoke, how many cigarettes do you smoke in a day?
- If you smoke, how long have you been smoking?
- Do you drink alcohol?
- Is your sense of smell keen?
- Do some odours bother you?
- Please state the odours that bother you
- Do you feel sick or did you feel sick in any vehicle when you were a child (including inability of reading in a vehicle)?

- Are you allergic to anything?
- Please state what you are allergic to
- Do you have asthma diagnosed by a physician?
- Is there any family member who has headache complaint?
- Do you suffer from disturbing vertigo from time to time?
- What is your vertigo associated with?

  1. It happens during headache
  2. Independent from headaches
  3. Both
- Have you been diagnosed with depression to date or have you been treated for depression?
- Do you have epilepsy (seizure disorder)?
- Do you have high/hypertension or do you use any medication for blood pressure?
- Do you currently (in last 1 year) have headache complaint?
- Have you ever had recurrent chronic headache in any period of your life?
- Have you ever seen/consulted a physician for your headache complaint?
- What was the specialty of the physician you consulted for headache?
- What was the diagnosis of this physician (the first one you consulted)?
- Have you ever seen/consulted another physician for headache afterwards?
- What were the specialties of these physicians?
- Have you been given different diagnoses by different physicians you consulted?
- What were these diagnoses?
- Do you use any medication for headache?
- Who recommended a medication for headache?

  1. Physician
  2. Pharmacist
  3. Relative/friend
  4. Self
  5. Other
- If a PHYSICIAN recommended, which medications were recommended?
- If a PHARMACIST recommended, which medications were recommended?
- If a RELATIVE/FRIEND recommended, which medications were recommended?
- If SELF-prescribing, which medications are taken?
- Are your headaches limiting your ability to work or enjoy life or do they bother you so as to consult a physician?

- Which of the following have you suffered during your headaches in last 3 months?

  1. Nausea or discomfort
  2. Light sensitivity
  3. Your headaches limited your ability to work or do what you need for at least one day
  4. None of above

- Do you feel that your headache is about to start before it starts (except aura)?
- Do you have any sensitivity on your scalp when you have headache? For example, can you comb your hair easily or does wind or touching hurt your scalp?
- Do you menstruate?
- Is your headache likely to start during your menstruation (from 3 days before menstruation to five days after it starts)?
- Is the headache you have during your menstruation different from other headaches or is it similar?
- Do you have headaches except your menstrual period?
- Have you ever given birth? If so, how many times?
- Was there any change in your headaches during your pregnancy?
- Have you ever used/do you use birth control pills?
- Was there any change in your headaches while you use birth control pills?
- Have you gone through menopause?
- Have your headaches changed during the period of menopause?
- How long have you been suffering from headaches?
- Since what age do you suffer from headache?
- Please specify the type of your headache

  1. In attacks
  2. Constantly
  3. Constantly but worsening from time to time
  4. In clusters
  5. Other

- What is the monthly frequency of your headache/how often or frequent do you have headache attack?
- How long is the average period of each headache (each attack) (without taking medication)?
- How many days in a month do you have headache?
- Which part of your head do you feel the headache the most (i.e. right side, left side or both sides, in other words, all your head)?

  1. Headache is limited with one side of the head
  2. On both sides but more intense on one side
  3. Same on both sides/all head

- Do you feel pain on your nape when you have headache?

- Please specify the character of your headache

  1. Pulsating
  2. Compressive
  3. Sensation of pressure
  4. Other (piercing, shocking, stinging etc.)

- Does your headache intensify when you move or lean forward and stand up?
- Which of the following discomforts do you feel with headache?

  1. Nausea
  2. Vomiting
  3. Light sensitivity
  4. Sound sensitivity
  5. Smell sensitivity
  6. Other
  7. None of above

- How intense is your headache in general?

  1. Mild pain not impairing your daily activities
  2. Moderate pain partially impairing your daily activities
  3. Intense pain impairing all your daily activities

- Please state the factors triggering your headache
- Which of the followings do you have before your headache starts?

  1. Sound, smell or light sensitivity
  2. Restlessness, nervousness
  3. Attention deficit/loss of concentration
  4. Increased urination
  5. Sugar craving
  6. Loss of appetite
  7. Yawning/shivers
  8. Other
  9. None of above

- AURA: Do you have symptoms such as blind spots, seeing flashing lights or lines, impaired speech, numbness of the face or part of the body, vertigo, double vision or weakness, starting immediately before or concurrently with headache and lasting 5 min to 1 h?
- Please indicate aura types:

  1. Visual Aura
  2. Sensory Aura
  3. Impaired speech
  4. Vertigo
  5. Loss of strength

- Please specify the DIAGNOSIS of headache:
  1. Migraine without aura
  2. Migraine with aura
  3. Frequent episodic tension-type headache
  4. Infrequent episodic tension-type headache
  5. Chronic tension-type headache
  6. Medication overuse headache
  7. Cluster headache and other short-lasting headaches
  8. Other
- Do you take any medication to stop the attack during your headache attacks?
- Which medications do you currently use during at attack for ATTACK TREATMENT?
- How many days in a month do you currently take painkillers?
- How many tablets in a day do you currently take painkillers?
- How long have you been using painkillers such frequently?
- (NOTE: This question will be asked to the subjects who take painkiller at least every 3 days or longer than 10 days in a month.) How long have you been using painkillers longer than 15 days in a month?
- Do painkillers relieve your pain?
- Are you pleased with painkillers?
- Please indicate the medications you like the most
- What are your expectations from the medication you take to relieve your pain (analgesics, ergos, triptans)?
- Do you receive any preventive or protective TREATMENT for headache other than attacks?
- Which of the following medications do you use regularly for prophylaxis treatment of headache?
- Is there any prophylaxis medication you have taken for longer than 1 month for headache?
- Please indicate the prophylaxis medications you have previously taken for headache
- How do you procure these medications?
  1. From state insurance system
  2. From private health insurance
  3. I pay myself (without reimbursement)
- Do you have medical board report for these medications?
- Do you have difficulty in buying these medications?
- Which methods have you used for treatment of your headache other than using medications?
- How do you follow the news/programs related with headache?
- Do you care about such news?

- Do these news inform or guide you?
- Does your headache destroy your quality of life?
- Have you suffered from economical loss because of your headache?
- Have you ever experienced issues in your family because of your headache?
- Have you ever experienced issues with your friends because of your headache?
- Have you ever experienced issues at your work or have you ever taken sick leave because of your headache?
- Have you ever experienced issues at your school or have you ever missed a day at school because of your headache?
- How many days did you miss at work or school in last 3 months because of headache? (WORK means the job you are paid for and SCHOOL means high school or university)
- What is the number of days when your performance at work or school reduced by half or more in last 3 months because of headaches? (Please do not include the days you missed)
- How many days could not you do housework in last 3 months because of headache? (HOUSEWORK covers working at home, repair and maintenance of house, shopping, care of children or relatives etc.)
- What is the number of days when your performance related with housework reduced by half or more in last 3 months because of headaches? (Please do not include the days you could not do housework)
- How many days could not you spare time to your family, social life or leisure time activities in last 3 months because of headaches?
- Comments of the Physician
